# To Determine the Diagnostic Accuracy of Diffusion-Weighted Imaging in the Diagnosis of Prostate Carcinoma Taking Histopathology As the Gold Standard

**DOI:** 10.7759/cureus.19508

**Published:** 2021-11-12

**Authors:** Kamran Siddiqui, Kausar Magsi, Junaid Iqbal, Anwar Ahmed, Adnan Fazal, Irfan Siddiqui, Shahmeer Khan

**Affiliations:** 1 Radiology, Aga Khan Health Service, Pakistan, Karachi, PAK; 2 Radiology, Sindh Institute of Urology and Transplantation, Karachi, PAK; 3 Radiology, Aga Khan University Hospital, Karachi, PAK; 4 Radiology, The Aga Khan University, Karachi, PAK; 5 Cardiology, National Institute of Cardiovascular Diseases, Karachi, PAK; 6 Emergency Department, Ziauddin University, Karachi, PAK

**Keywords:** histopathology, digital rectal examination (dre), diffusion weighted imaging (dwi), prostate-specific antigen (psa), prostate cancer

## Abstract

Introduction

Carcinoma of the prostate is the most common malignancy among males. Serum prostate-specific antigen (PSA) levels and digital rectal examination (DRE) are the initial investigations for the detection of prostate cancer. In recent years, some investigators have used color Doppler ultrasound and diffusion-weighted imaging (DWI) for the diagnosis of prostate cancer and avoided invasive and painful investigation, i.e., biopsy. The purpose of the study is to determine the validity of DWI in detecting prostatic cancer taking histopathology as the gold standard.

Material and methods

This cross-sectional study was conducted prospectively in the radiology department of a tertiary care hospital from January 1, 2019, to December 31, 2020. This study was approved by the Departmental Research Committee. A total of 272 male patients were included in our study who have elevated PSA levels (>4.0 ng/ml) with symptoms of hematuria and urinary retention. All included subjects were sent to the radiology department for DWI imaging. The DWI imaging was analyzed for prostate cancer and the results were correlated with histopathological diagnosis.

Results

The average age of patients was 50.28±9.93 years. The sensitivity, specificity, positive predictive value, negative predictive value, and accuracy of DWI in the diagnoses of prostate cancer were 86.7%, 87.8%, 75.8%, 93.8%, and 87.5%, respectively.

Conclusion

DWI is an informative and non-invasive imaging modality with high diagnostic accuracy for the diagnosis of prostate carcinoma.

## Introduction

Prostate cancer is the most common malignancy among males [1,2]. It has now become the third most common cancer in men. The incidence has increased to half a million new cases per year [3], contributing nearly 10% of all cancers in men [2].

Prostate cancer is the second leading cause of cancer death following lung cancer [4]. Although the incidence and mortality rates vary among countries, it has been reported that the incidence of the disease has increased in many countries since the early 1990s [5]. The most common site of malignant transformation in the prostate gland is the peripheral zone (70%) followed by the central zone (15-20%) and transitional zone (10-15%) [3,6].

Prostate cancer can be treatable if detected early resulting in a reduction of cancer mortality [1,7]. The investigations for the detection of prostate cancer are serum prostate-specific antigen (PSA), digital rectal examination (DRE), transrectal ultrasonography (TRUS), magnetic resonance imaging (MRI), and transrectal prostate biopsy. Serum PSA and DRE are conventional non-invasive investigations. In recent years, some investigators have used color Doppler ultrasound and diffusion-weighted imaging (DWI) for the diagnosis of prostate cancer and avoided an unnecessary biopsy [5].

Recently, DWI has emerged as the non-invasive imaging modality with high diagnostic accuracy in determining the size, extent, and location of cancerous lesions in the prostate gland [1,8,9].

Various studies showed that DWI is an important imaging modality in the detection of tumors in different regions like the breast, prostate, urinary bladder, cervix, colon, ovary, pancreas, and liver. DWI also helps in the characterization of tumors as malignant tumors show more diffusion restriction and much lower apparent diffusion coefficient (ADC) levels than benign tumors due to their increased cellularity [10].

Various studies have examined the diagnostic accuracy of DWI in detecting prostate cancer with wide ranges of sensitivity and specificity (29-94% and 39-100%, respectively) [4]. In 19 publications, a total of 5,892 lesions were analyzed, and results reported that the sensitivity and specificity in the diagnosis of prostate cancer were 69% and 89%, respectively [9].

It is observed that the literature shows different sensitivity and specificity values for DWI, for which we would like to determine the values in our population that can increase diagnostic confidence and thus improve the overall management of this highly prevalent and aggressive disease.

## Materials and methods

This cross-sectional study was conducted prospectively in the radiology department of Sindh Institute of Urology and Transplant (SIUT), a tertiary care hospital from January 1, 2019, to December 31, 2020. This study was approved by the Departmental Research Committee. A total of 272 male patients were included in our study of age between 35 and 80 years who have elevated PSA levels (>4.0 ng/ml) with symptoms of hematuria and urinary retention. All included subjects were sent to the radiology department for DWI after getting informed consent. Patients with prior history of prostate cancer, hormonal, radiation, or surgical treatment were excluded from this study. The DWI imaging was analyzed for prostate cancer and the results were correlated with histopathological diagnosis.

DWI was performed on 1.5 Tesla Magneton Harmony (SIEMENS, Germany) with a dedicated prostate coil. The images were studied and reported by at least two trained and qualified radiologists with minimum five years of post-fellowship clinical experience. All patients underwent transrectal biopsy for histopathological diagnosis. A proforma was used to record patients’ demographics like name, age, hospital registration number, height, weight, BMI, duration of symptoms, prostate cancer on DWI as positive or negative, and prostate cancer on histopathology as positive or negative.

Patient data were compiled and analyzed through the Statistical Package for Social Sciences (SPSS) version 21 (IBM Corp., Armonk, NY). Frequency and percentage were computed for qualitative variables like symptoms, findings of DWI, and findings of histopathology.

Mean±SD was calculated for quantitative variables, i.e., age and PSA level. Sensitivity, specificity, positive predictive value, negative predictive values, and the diagnostic accuracy of DWI were calculated taking histopathology as the gold standard by using 2 × 2 tables.

Stratification with respect to age, symptoms, and PSA was done. Post-stratification diagnostic accuracy, sensitivity, specificity, PPV, and NPV were also be calculated.

## Results

**Figure 1:**
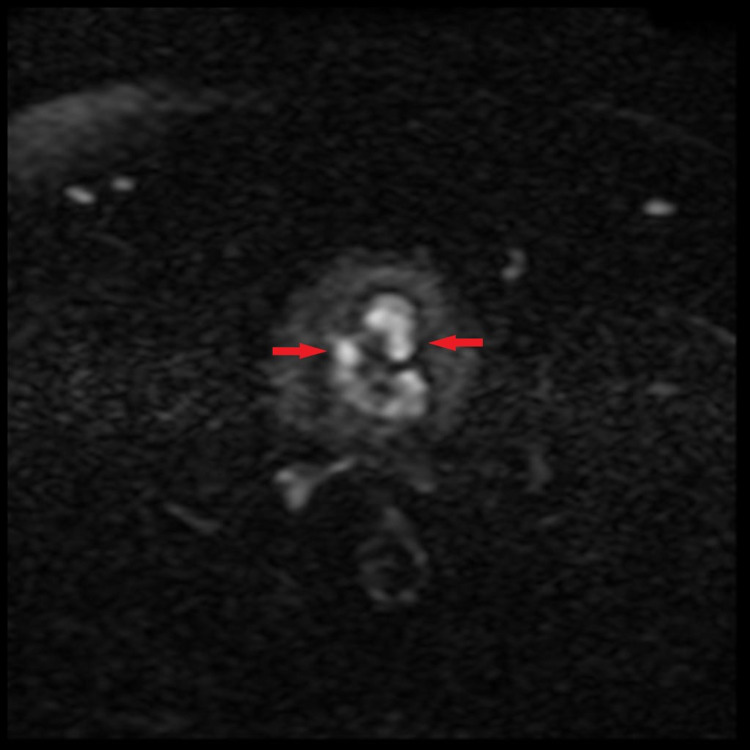
DWI sequence showing diffusion restriction (high signals) within prostate gland (arrows) consistent with prostatic carcinoma. DWI: diffusion-weighted imaging.

**Figure 2:**
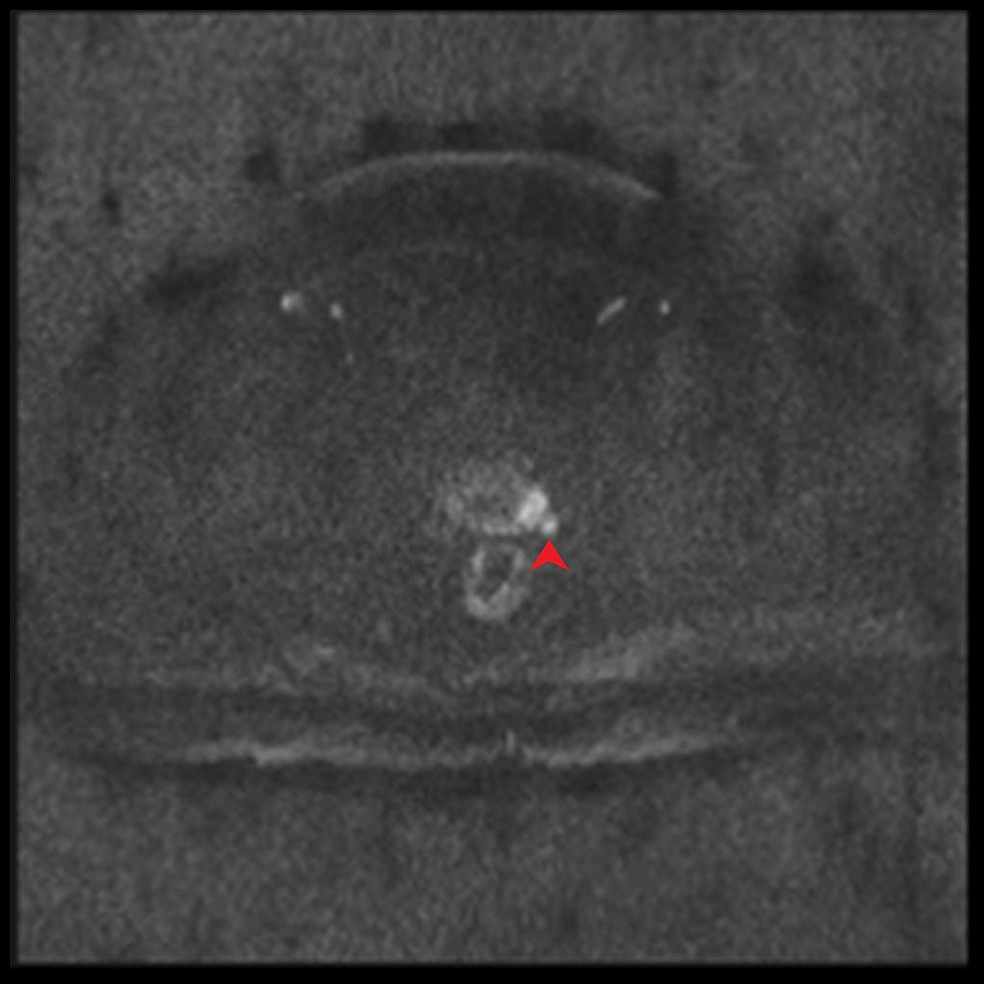
DWI sequence showing prostatic carcinoma with an extracapsular extension on the left side (arrowhead). DWI: diffusion-weighted imaging.

**Figure 3:**
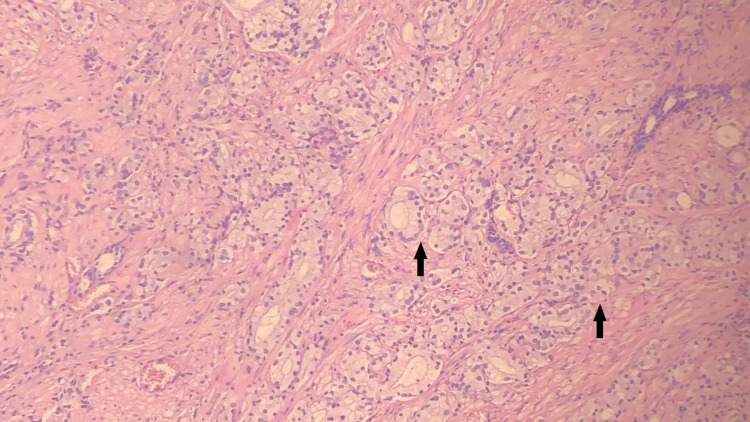
Prostate adenocarcinoma shows cribriform and fused closely packed glands in core biopsy specimen from prostate (arrows) Gleason grade 4 + 4 = score of 8, with hematoxylin and eosin staining, ×100.

**Figure 4:**
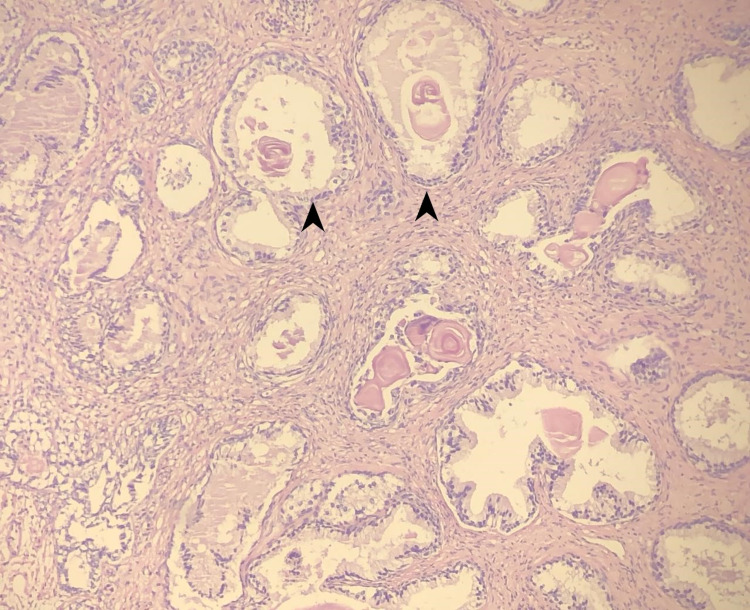
Biopsy specimen of prostate gland lined by cuboidal secretory cells, with pale cytoplasm, regular nuclei (arrowheads) with variably sized glands representing benign prostatic hyperplasia with hematoxylin and eosin staining, ×100.

There were 272 male patients who have elevated PSA levels (>4.0 ng/ml) and with symptoms of hematuria and urinary retention were included in this study (Table 1). Most of the patients were between 51 and 60 years of age. The average age and PSA were 50.28±9.93 years and 7.23±1.64 ng/ml, respectively. Out of 272 cases, hematuria was observed in 71.32% cases and urinary retention was 80.88%. Out of 272 cases, 95(34.93%) were positive by DWI for prostate cancer (Figures 1 and 2 show DWI of prostate carcinoma and extracapsular extension of cancer, respectively), while 83(30.51%) were confirmed positive by histopathology (Table 2). Figures 3 and 4 show histopathology of prostate cancer and benign prostate hypertrophy, respectively.

**Table 1:** Frequency and percentages of symptoms.

Symptoms	With hematuria	Without hematuria	With urinary retention	Without urinary retention
Frequency	78	194	52	220
Percentage	28.68%	71.32%	19.12%	80.88%

**Table 2:** Findings of DWI and histopathology for prostate cancer. DWI: diffusion-weighted imaging.

Findings	On DWI	On Histopathology
Positive	95 (34.93%)	83 (30.51%)
Negative	177 (65.07%)	189 (69.49%)

The sensitivity, specificity, positive predictive value, negative predictive value, and diagnostic accuracy of DWI in diagnosing prostate cancer were 86.7%, 87.8%, 75.8%, 93.8%, and 875%, respectively, as shown in Table 3. Diagnostic accuracy of DWI in diagnosing prostate cancer was also observed with respect to age, PSA levels, and urinary symptoms as shown in Table 4.

**Table 3:** Diagnostic accuracy of diffusion-weighted imaging for prostate cancer. NPV: negative predictive value, PPV: positive predictive value.

Sensitivity	Specificity	PPV	NPV	Diagnostic accuracy
86.7%	87.8%	75.8%	93.8%	87.5%

**Table 4:** Diagnostic accuracy of DWI for prostate cancer with respect to age, PSA levels, and urinary symptoms. NPV: negative predictive value, PPV: positive predictive value, DWI: diffusion-weighted imaging, PSA: prostate-specific antigen.

	Sensitivity	Specificity	PPV	NPV	Diagnostic accuracy
Age <50 years	84.8%	90.1%	75.5%	94.3%	88.7%
Age >50 years	88.0%	85.7%	75.9%	93.3%	86.5%
PSA 5 to 7	90.7%	86.8%	73.6%	95.8%	87.9%
PSA > 7	82.5%	89.2%	78.6%	91.4%	86.9%
Symptoms with hematuria	91.8%	85.7%	74.7%	95.8%	87.6%
Symptoms with urinary retention	85.1%	87.6%	75%	93.1%	86.8%

## Discussion

Prostate cancer is not only the commonest cancer but also the leading cause of cancer-related death in men. The growing elderly population has resulted in the largest increase in the number of new cases of prostate cancer [11]. For proper management and complete cure, tumor localization along with early detection and staging is very important [12].

Functional anatomy and molecular imaging information plays important role in the more accurate characterization of disease [13]. In the early stage of prostate cancer, MRI can be more helpful in the identification of the disease [14]. Serum PSA level and TRUS-guided biopsy are performed for the histopathological diagnosis prior to surgical resection but the high false-negative rate of TRUS-guided biopsy is the main challenge in further management [15] along with poor tolerance of patients for invasive procedures [16]. To overcome these challenges, a non-invasive method with high accuracy for the diagnosis is the bailout and MRI prostate is the modality of choice in the stated scenario because of its dynamic contrast studies along with extensive sequences and spectroscopy [17]. Overdiagnosis and overtreatment remain the main challenges along with missed tumors in the anterior and apical parts of the prostate gland [18]. It is very difficult to locate tumors in the transition zone [19].

DWI measures the spontaneous movement of water molecules known as Brownian motion and is highly dependent on the cellular environment of the water. The degree of movement of water molecules is called diffusion. Since diffusion is mainly restricted by cell membranes, the degree of restriction of freedom of movement is proportional to the cell density of a tissue. Single-shot echo-planar-imaging spin-echo sequences usually apply for DWI with an application of two gradient pulses of equal strength. The strength of the gradient pulse is expressed in terms of the b value. Two b-values should be obtained for the calculation of ADC maps; in routine practice usually, three b-values ​​are obtained, one low (50 s/mm^2^), one intermediate (400 s/mm^2^), and one high value (1,000 s/mm^2^). Diffusion is hampered in areas with densely packed tumor cells which appear bright on DWI and dark on the ADC map [20]. ADC values are usually lower in malignant lesions than in benign lesions of the prostate gland [21]. DWI is highly sensitive to detecting malignant lesions in the peripheral zone of the prostate gland than in the transitional zone [22]. b-Values are the most important factor for tumor detection, Metens et al. in their study showed that the maximum malignant lesions detected by using high b-values (1,500 s/mm^2^) also give a high contrast-to-noise ratio [23]. DWI and ADC mapping not only detect tumors accurately but also detect extracapsular extension and seminal vesical involvement of tumor [24].

A study on 283 patients in which 39 patients had a seminal vesicle infiltration of the tumor showed significantly lower ADC values as compared to seminal vesicles that were tumor-free [25].

The incidence and mortality of prostate cancer are much more common in older men with an average age of 66 years at the time of diagnosis [26]. In our study, most of the patients were between 51 and 60 years of age; the average age and PSA were 50.28±9.93 years and 7.23±1.64 mg/ml. In patients with prostate carcinoma, the frequency of Hematuria is unknown, as it can be due to the presence of the tumor itself, could be secondary to prior radiotherapy, or due to post-surgical stone formation. Out of 272 cases in our study, hematuria was observed in 71.32% of cases. Sometimes the prostate cancer can encase or compress the urethra causing significant post-void urinary retention, 80.88% of patients in our study were having urinary retention.

In various studies and meta-analyses, articles showed wide ranges of sensitivity and specificity of DWI for the detection of prostate carcinoma ranging from 29% to 94 % and 39% to 100%, respectively [27]. In our study on the local population with a large sample size of 272 cases, the sensitivity and specificity were found out to be 86.7% and 87.8%, respectively, with high diagnostic accuracy.

Various magnetic resonance imaging sequences like T2-weighted image, DWI, dynamic contrast-enhanced MRI, and MR spectroscopy can be used for the diagnosis of prostate carcinoma; however; they also carry some limitations [28]. Due to the high diagnostic accuracy of DWI, this should be considered and adopted as one of the main sequences of MRI for the detection of prostate carcinoma.

## Conclusions

In conclusion, our study showed that DWI plays an important role in the diagnosis of prostate cancer, as it provides substantial information regarding tumor characterization, localization, extension, local infiltration, staging, and response assessment with high diagnostic accuracy. DWI can also help in the assessment of early metastatic spread to take an early decision for metastatic-directed therapy. It may also help in the detection of lesions in occult or difficult areas like in the transitional zone. The advantages of DWI are its non-invasiveness, no need for intravenous contrast, radiation exposure or additional hardware and imaging coils, short imaging time, and very simple post-processing techniques. Optimization of image acquisition techniques and interpretation is required for further application of DWI.
